# Evaluation of Macular Thickness Changes after Uncomplicated Phacoemulsification Surgery in Healthy Subjects and Diabetic Patients without Retinopathy by Spectral Domain OCT

**DOI:** 10.3390/diagnostics12123078

**Published:** 2022-12-07

**Authors:** Mikel García Gómez de Segura, Ana Martín-Arroyuelos, Isabel Pinilla, Javier Araiz

**Affiliations:** 1 Department of Pharmacology and Physiology, Zaragoza University, 50009 Zaragoza, Spain; 2 Instituto Clínico Quirúrgico de Oftalmología (ICQO), 48010 Bilbao, Spain; 3 Department of Quantitative Methods, University of the Basque Country, 48015 Bilbao, Spain; 4 Department of Ophthalmology, Hospital Clínico Universitario, 50009 Zaragoza, Spain; 5 Department of Surgery, Zaragoza University, 50009 Zaragoza, Spain; 6 Institute for Health Research, Aragon (IIS Aragon), 50009 Zaragoza, Spain; 7 Department of Ophthalmology, University of the Basque Country, 48940 Bilbao, Spain

**Keywords:** OCT, macular thickness, phacoemulsification, diabetes mellitus, diabetic retinopathy, macular edema

## Abstract

Purpose: To assess differences in the evolution of macular thickness after uncomplicated phacoemulsification surgery between non-diabetic subjects and patients with diabetes mellitus (DM) without diabetic retinopathy (DR), using Spectral Domain OCT (SD-OCT). Methods: We performed a unicentric prospective study including one hundred and thirty-one eyes of 70 patients divided into two groups—34 well-controlled DM patients without DR and 36 non-diabetic subjects—who underwent phacoemulsification for cataract surgery. Eyes that developed pseudophakic cystoid macular edema (PCME) were excluded from the study, leaving us with 64 patients. Macular thickness was analyzed using Cirrus HD-OCT (Macular Cube 512 × 128 protocol) preoperatively and on postoperative days 7, 30, 90, and 180. For cases with information available for both eyes, one eye was randomly selected for analysis. Results: A total of 64 eyes from 64 patients were analyzed in this study. The mean value of HbA1c in the diabetic group was 7%. After uncomplicated cataract surgery, patients showed no increase of the foveal, parafoveal, and perifoveal retinal thickness on postoperative day 7. However, thickness values increased on days 30, 90, and 180 after surgery in both groups, and peak at 90 days. There was no difference in macular thickness before or after surgery between DM and non-diabetic patients (*p* = 0.540). Conclusion: Macular thickness increases up to 6 months after uncomplicated cataract surgery in both DM patients without DR and non-diabetic subjects, with no differences between increases in both groups.

## 1. Introduction

Diabetes mellitus (DM) is the most frequent metabolic disorder in humans. It is one of the greatest public health problems worldwide, involving a large amount of financial and human resources. It is estimated that there will be 439 million DM people in the world by the year 2030 [1].

Cataract development has a higher incidence and faster progression in DM patients [2,3]. The most frequent type in these patients is the nuclear one [4]. Current cataract surgery techniques have managed to considerably reduce the risk of inflammatory complications in these patients, particularly diabetic macular edema (DME) [5], although cataract surgery is still considered a high-risk surgery in DM patients compared to subjects without comorbidities [6].

Macular edema (ME) is the leading cause of poor visual acuity (VA) after cataract surgery in DM patients [7], due to either pseudophakic cystoid ME (PCME), also known as Irvine–Gass syndrome, or the progression of diabetic maculopathy, which is as a result of pre-existing DME at the time of surgery.

The risk factors that influence increase in macular thickness after surgery include presurgical DME [5], the DR stage [8,9], the glycated hemoglobin (HbA1c) level [10], the use of insulin [11], the duration of diabetes [12], ocular hypertension [13], and hyperlipidemia [14].

Some authors affirm that DM patients have an altered blood–retinal barrier even in the absence of obvious signs of DR, and this could justify higher rates of ME after cataract surgery [15,16,17]. However, the incidence of this worsening is not clear yet [18].

It is unknown whether the progression of ME in DM patients after cataract surgery is a consequence of the surgical trauma or a reflection of the natural progression of the disease, but it seems to be related to the type of diabetic population (stage of the disease or metabolic control). This study focuses on DM patients without signs of DR or DME, who are commonly seen in practice.

Optical coherence tomography (OCT) is an objective, non-invasive, well-tolerated, non-contact method for quantitative measurements of retinal thickness, with high reproducibility [19,20].

OCT has substantially boosted the sensitivity of detection of retinal thickening and ME compared to clinical examination and is as effective as fluorescein angiography (FA) [21,22].

The purpose of our study was to analyze changes in macular thickness after uncomplicated phacoemulsification surgery in non-diabetic subjects and DM patients without DR lesions, and to determine if DM increases the risks associated with these changes.

## 2. Materials and Methods

We performed a unicentric prospective study including a consecutive cohort of 36 DM patients without DR and 34 non-diabetic subjects diagnosed with age-related cataract. A total of 131 eyes were studied. The study was carried out at the Instituto Clínico Quirúrgico de Oftalmología (ICQO) in Bilbao, Spain between January 2019 and December 2019, with approval from the Institutional Ethics Committee. CEIC E18/43, clinical research ethics committee from the Hospital Universitario Cruces, Spain.

The inclusion criteria were DM without DR and non-diabetic subjects with cataract. The exclusion criteria were a previous history of intraocular surgery, uveitis, glaucoma or any vitreoretinal pathology, a refractive error with a spherical equivalent (SE) over 5.5 diopters (D) or astigmatism higher than 3D, and any intraoperative or postoperative complication. To ensure a correct analysis of macular thickness, OCT scans with a signal strength inferior to 6 in the preoperative exam, were excluded from the study.

Each subject’s complete history was obtained, including age, sex, and duration, as well as the DM type and HbA1c level for the diabetic group. Preoperatively, ophthalmic examination included measurement of refractive errors, BCVA with Snellen charts, and intraocular pressure (IOP) with Goldman tonometry, as well as biometry with IOL-Master (Carl Zeiss Meditec, Dublin, Ca, USA), slit-lamp biomicroscopy posterior segment examinations, and spectral domain (SD) OCT. Patients were followed up at postoperative days 1, 7, 30, 90, and 180.

OCT scan was performed with Cirrus HD-OCT 4000 (Carl Zeiss Meditec, Dublin, CA, USA). Macular cube 512 × 128 protocol was used, with all scans having signal strength greater than 6 and no motion or blinking artefacts. The thickness measurements for each of the nine map sectors as defined by the Early Treatment Diabetic Retinopathy Study (ETDRS) and the average retinal thickness and total macular volume (MV) were generated by the Cirrus HD-OCT internal algorithm (software version: 6.9). The ETDRS grid includes the central value of the 1 mm diameter ring and 4 quadrants (superior, inferior, temporal, and nasal) in the inner or parafoveal (3 mm) and outer or perifoveal ring (6 mm). OCT measurements were done before surgery and in the defined postoperative times.

**Surgical procedure:**

Before surgery, mydriasis was achieved using 10% phenylephrine hydrochloride (Colicusí Fenilefrina^®^; M4 PHARMA, S.L., Barcelona, Spain), 1% cyclopentolate hydrochloride (Colicursí Ciclopejico^®^; Alcon Healthcare, S.A., Cornellà de Llobregat, Spain), and 0.5% ketorolac trometamol (Acular^®^; Allergan S.A., Tres Cantos, Spain). A 2% lidocaine gel anesthetic was applied on the ocular surface few minutes before surgery.

A lid speculum was used to hold the eyelids apart. The ocular surface was irrigated with 5% povidone iodine for 30–60 s to wash out debris and particles from the conjunctival fornices, followed by generous washing with balanced salt solution to remove all traces of antiseptic solution from the surgical field.

A 15° stab knife was used to fashion a small paracentesis at the peripheral cornea 3–4 clock hours before the main surgical incision. Intracameral injections of anesthetic (lidocaine 2%) and viscoelastic filling of the anterior chamber were performed after making the side ports.

After making a 2.4 mm clear corneal tunnel incision, forceps were used to create a continuous curvilinear capsulorrhexis, followed by hydrodissection, hydrodelineation, and nucleus rotation, posterior chamber phacoemulsification, and foldable acrylic IOL implantation within the capsular bag.

At the end of the surgery, 0.1 mL of 10 mg/mL cefuroxime was injected in the anterior chamber, followed by wound leak testing.

After surgery, all patients received tobramycin 1 mg/mL and dexamethasone 3 mg/mL (Tobradex^®^; Novartis Farmacéutica, S.A., Barcelona, Spain) and 0.5% ketorolac trometamol (Acular^®^; Allergan S.A., Tres Cantos, Spain) four times a day for at least two weeks. All the surgeries were performed by the same surgeon. All surgical procedures were performed with the same equipment (Alcon Constellation, Fort Worth, TX, USA). There were no significant differences regarding energies used.

**Statistical Analysis:**

All variables were collected in the Excel 2016 program and the information was subsequently analyzed using SPSS Statistics for Windows, Version 26.0 (SPSS, IBM Corp, Armonk, NY, USA). *p*-values less than 0.05 were considered significant.

From the initial cohort of patients, eyes that developed PCME were excluded. In patients that underwent surgery for both eyes, one eye was randomly selected for analysis.

A repeated measures MANOVA (RMM) was used to assess whether diabetes or gender had a significant effect on the temporal evolution of different variables considered as a whole and whether there were significant differences between the values of these variables measured at different times.

If diabetes or gender was detected as an influencing factor, analysis of variance was performed for each variable separately to detect in which of them there were significant differences between groups (diabetics vs. non-diabetics or men vs. women). Paired t-tests were also performed to evaluate the differences between the time points considered in the analysis. In these cases, all *p*-values were adjusted by the Bonferroni correction.

Differences in macular thickness between operated and unoperated eyes were studied in a group of 18 patients to compare the temporal evolution of both eyes. Wilcoxon tests were performed, with the *p*-values adjusted by the Bonferroni correction.

To determine if there is a relationship between BCVA and the macular thickness obtained at different moments in time, the *p*-values of the corresponding Pearson correlation coefficients, adjusted by the Bonferroni correction, have been obtained. To assess if gender or diabetes have a significant effect on the temporal evolution of BCVA, a repeated measures ANOVA (RMA) has been performed.

## 3. Results

Seventy patients (39 females, 56%; 31 males, 44%) were initially included in this study. The average age of the patients was 71.66 ± 1.95 years (range: 47 to 86 years). The DM group was composed of 36 patients (51%) and the control group included 34 non-diabetic subjects with age-related cataract (49%). The mean value of HbA1c in the DM group was 6.99%. There were no differences between surgeries and the used energy.

After exclusion of eyes that developed PCME and randomization in patients with information about both eyes, a total of 64 eyes from 64 patients (33 females, 52%; 31 males, 48%) were analyzed. Thirty-two of these patients (50%) were DM patients and thirty-two were non-diabetic subjects (50%). The average age of the patients was 71.66 ± 2.02 years (range: 47 to 85 years). The mean value of HbA1c in the group of diabetic patients was 7%.

In both diabetic and non-diabetic patients, there was no significant change in the foveal, parafoveal, and perifoveal retinal thickness after phacoemulsification on postoperative day 7. There was increase in thickness on days 30, 90, and 180. OCT thickness values of the central 1 mm ring, the four quadrants (superior, inferior, nasal, and temporal) of the 3- and 6-mm ring, the MV and the average thickness are presented in Table S1.

**Figure 1:**
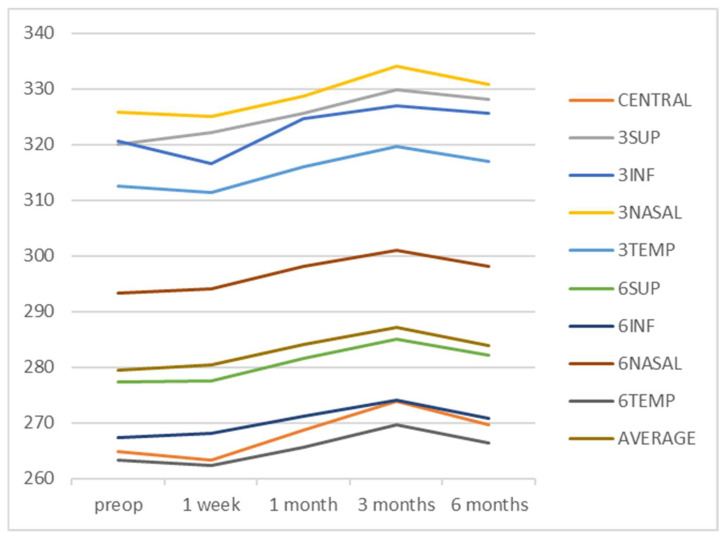
Temporal evolution of the mean macular thickness in the different ETDRS grid areas. The thickness measurements are expressed in microns. Abbreviations: CENTRAL is the central 1 mm ring; 3SUP, 3INF, 3NASAL, and 3TEMP are, respectively, the superior, inferior, nasal, and temporal quadrants of the 3 mm ring in the ETDRS grid; 6SUP, 6INF, 6NASAL, and 6TEMP are, respectively, the superior, inferior, nasal, and temporal quadrants of the 6 mm ring in the ETDRS grid; MV is the macular volume and AVERAGE is the average retinal thickness.

Temporal evolution of the mean macular thickness in the areas of the ETDRS grid was evaluated and is presented in Figure 1.

Almost all the studied areas showed changes compared to the preoperative measurements. Table 1 shows the temporal differences for each of the areas of the ETDRS.

**Figure 2:**
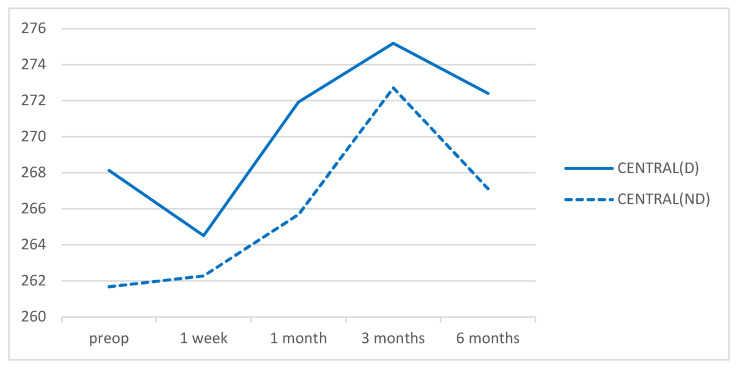
Mean values of the retinal thickness in the central area of the 1 mm diameter ring for DM patients and non-diabetic subjects during the follow-up time points. Values are expressed in microns.

**Figure 3:**
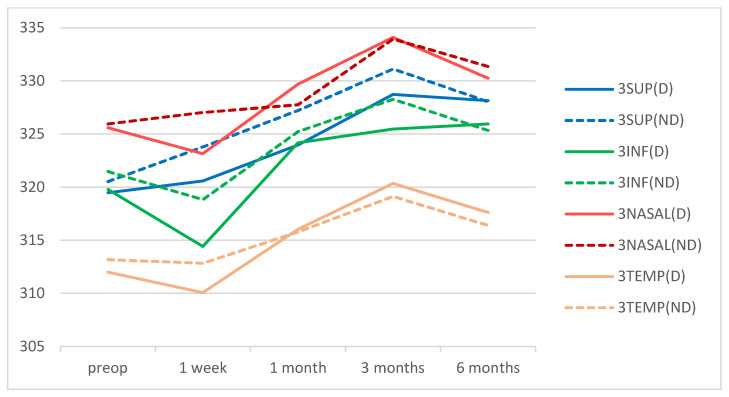
Mean values of the retinal thicknesses in the 3 mm ring of the ETDRS macular grid for DM patients and non-diabetic subjects during the follow-up time points. Values are expressed in microns.

**Figure 4:**
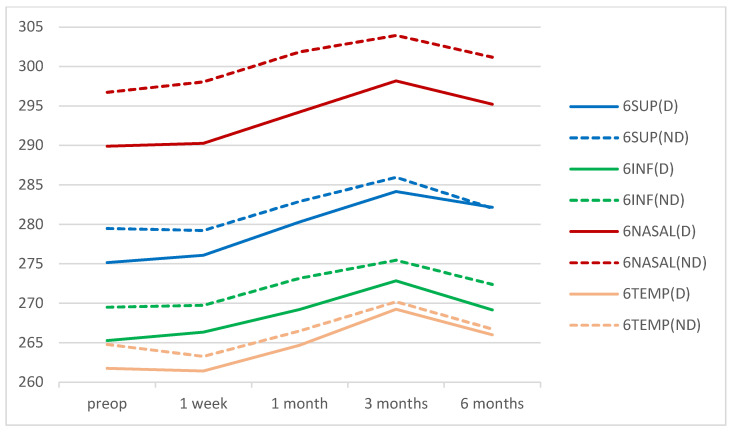
Mean values of the retinal thicknesses in the 6 mm ring of the ETDRS grid for DM patients and non-diabetic subjects during the follow-up time points. Values are expressed in microns.

There were no significant differences in macular thicknesses between eyes of the DM group and eyes of the control group at the preoperative time point nor at any of the postoperative points in the central area. There were also no significant differences in the mean thickness values of the para- or perifoveal rings between the two groups preoperatively nor at any postoperative time point (Figure 2, Figure 3 and Figure 4). The acronyms in these figures are CENTRAL: the central 1 mm ring; 3SUP, 3INF, 3NASAL, and 3TEMP are, respectively, the superior, inferior, nasal, and temporal quadrants of the 3 mm ring in the ETDRS grid; 6SUP, 6INF, 6NASAL, 6TEMP are, respectively, the superior, inferior, nasal, and temporal quadrants of the 6 mm ring in the ETDRS grid; MV: the macular volume and AVERAGE: the average retinal thickness. In parentheses: D: diabetic group; ND: non-diabetic group.

**Figure 5:**
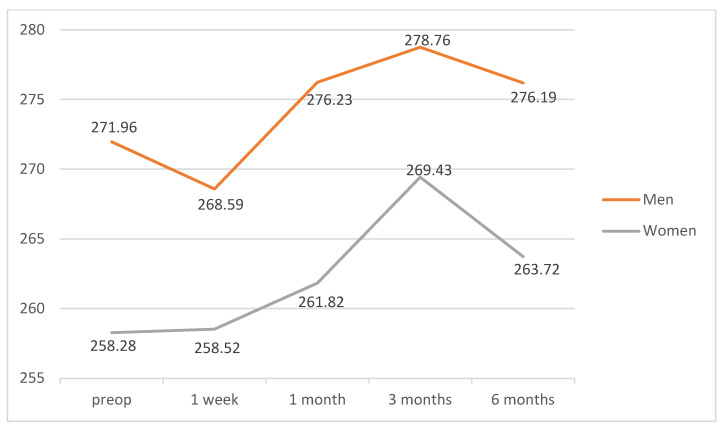
Mean values of the retinal thicknesses in the central 1 mm ring of the ETDRS grid for DM patients and non-diabetic subjects grouped by gender during the follow-up time points. Values are expressed in microns.

Analyzing gender as an influence factor, statistically significant differences were found in the central area of the ETDRS grid (*p* = 0.035), with thicker values in men than in women. There were no differences in the other areas of the ETDRS. Data are shown in Figure 5.

Differences between macular thickness in operated and unoperated eyes were studied in a group of 18 patients, using the unoperated eye as the control. Two of these patients later developed PCME and were excluded, leaving 16 patients for the final analysis (Table 2). Non-parametric tests were used for the analysis (Wilcoxon tests with *p* values adjusted by the Bonferroni correction).

**Figure 6:**
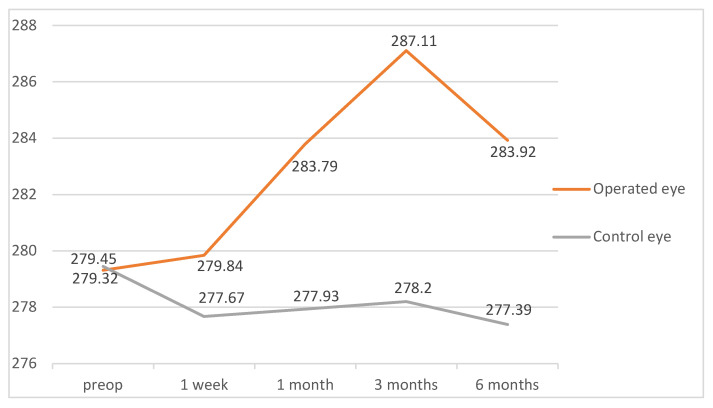
Mean values of average retinal thickness for operated and control eyes during the follow-up time points. Values are expressed in microns.

**Figure 7:**
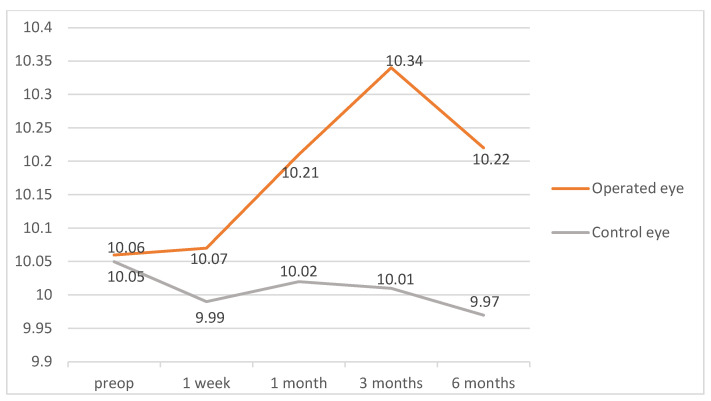
Mean values of macular volume for operated and control eyes during the follow-up time points. Values are expressed in mm^3^.

There were significant differences in the macular thicknesses, with higher thickness values for the operated eye compared to the control one in some of the areas and at different follow-up time points. On postoperative day 7, no differences were found. On postoperative day 30, we found significant differences in the inferior quadrant of the 6 mm ring (*p* = 0.03). On day 90, we found significant differences in both the superior (*p* = 0.03) and nasal (*p* = 0.03) quadrants of the 3 mm ring, in the nasal quadrant of the 6 mm ring (*p* = 0.01), in the MV (*p* = 0.01), and in the average retinal thickness (*p* = 0.01). In the 180-day measurements, we found differences in the following areas: superior (*p* = 0.03), inferior (*p* = 0.04), and nasal (*p* = 0.01) quadrants of the 3 mm ring; inferior (*p* = 0.03) and nasal (*p* < 0.01) quadrants of the 6 mm ring; MV (*p* < 0.01); and average retinal thickness (*p* < 0.01). Figure 6 and Figure 7 show the temporal evolution of the average retinal thickness and MV measurements for operated and unoperated eyes. The unoperated eye showed no changes in macular thickness over time.

Regarding the relationship between visual acuity and macular thickness, the only significant correlation coefficient (*r* = 0.399, with *p* = 0.011) has been obtained for the values of BCVA and macular thickness of the superior quadrant of the 3 mm ring, obtained 30 days after surgery.

There were no significant differences in BCVA between diabetic and non-diabetic patients (*p* = 0.418), nor between men and women (*p* = 0.229).

**Figure 8:**
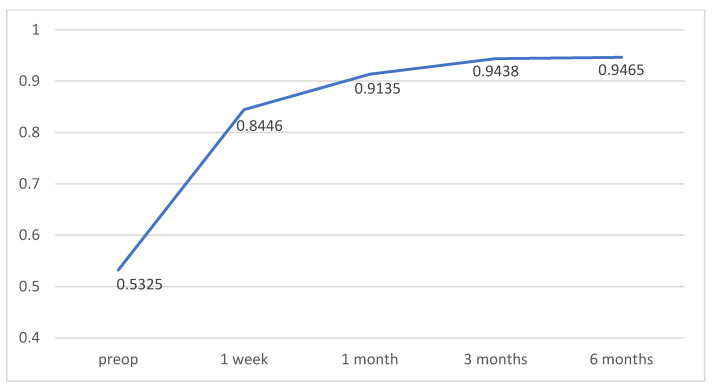
Temporal evolution of mean best corrected visual acuity (BCVA).

Temporal evolution of the BCVA is presented in Figure 8. It can be seen that, after the operation, the value of this variable increases throughout the entire period considered in the study.

## 4. Discussion

Although the pathogenesis of ME after cataract surgery is probably multifactorial and remains unknown, it appears to be associated with postoperative inflammation induced by prostaglandins and other inflammatory mediators [23]. DM patients have chronic microangiopathy, which makes them more prone to developing subclinical ME after cataract surgery [24].

Some studies have evaluated the chances of changes in macular thickness or developing ME after surgery in DM patients without DR compared to non-diabetics. Dennitson et al. [25] places the risk of developing DME at 1% in the first year after cataract surgery, and Chu et al. [26] at 1.8%.

The descriptive values obtained in this study coincide with the current normative data for Cirrus OCT [27,28,29].

In the present study, prior to surgery, no significant differences were observed in the macular thickness values between the DM group and the non-diabetic group. Some studies have reported similar values for DM patients with no DR lesions and normal subjects [30,31,32]. We also found no differences in macular thickness between the two groups after cataract surgery. Numerous studies have presented similar results, with DM without DR exerting no significant influence on macular thickness after uncomplicated cataract surgery [32,33,34,35,36].

However, other authors have found significant differences in macular thickness among patients after cataract surgery [31,37,38]. In our study, we excluded all patients that developed any degree of ME, which could explain the difference between our results and those of these authors.

In our study, we observed changes in retinal thickness during the studied time points. Preoperative values were similar to those found one week after surgery. One month after surgery, there was statistically significant increase in retinal thickness values that continued up to three months, but after six months, the values reduced to become similar to those obtained one month after the operation.

Increased subclinical macular thicknesses after uncomplicated cataract surgery are still unclear. Our results are similar to those published by Cagini et al. [39] and Ikegami et al. [33]^,^ who reported a peak in macular thickness 3 months after surgery. Prakash et al. [34] found a peak at 6 weeks, Perente et al. [40] and Bamahfouz et al. [41] at the one-month time point, like Katsimpiris et al. [31] in their control group. On the contrary, Giocanti-Auregán et al. [42]^,^ in diabetic patients without DR, observed a peak macular thickness value at 6 months, whereas Katsimpris et al. [31] did at 12 months. Part of these changes could be related to the use of topical steroids.

The diabetic and non-diabetic patients whose operated and control eyes were compared, we saw that the evolution of both eyes was different, which was expected. The non-operated contralateral eyes showed no changes in their thickness over time.

Regarding the relationship between BCVA and macular thickness, the only significant correlation coefficient has been obtained for the superior quadrant of the 3 mm ring, obtained one month after surgery.

These postoperative increases in retinal thickness were asymptomatic, probably reflecting changes in the permeability of the retinal–blood barrier, and may be part of the pathophysiology of the macular region in cataract surgery. They were similar for both DM without DR and non-diabetic patients. Other values such as retinal sensitivity provide by microperimetry would improve our knowledge of the macular status [43].

## 5. Conclusions

The present study has demonstrated a significant increase in macular thickness and volume up to 6 months after uncomplicated cataract surgery both in diabetic patients without DR and non-diabetic subjects. No significant differences were found between both groups. This postoperative inflammatory activity is subclinical and is the same in both groups. Good metabolic control is important to avoid a worse visual prognosis after cataract surgery.

## Figures and Tables

**Table 1:** *p*-values paired test of comparison between different time points for all variables. Differences with *p*-values < 0.05 are shown in shade.

Variable	Pre–7d	Pre–30d	Pre–90d	Pre–180d	7d–30d	7d–90d	7d–180d	30d–90d	30d–180d	90d–180d
CENTRAL	1.000	<0.001	0.004	0.012	0.018	<0.001	<0.001	0.337	1.000	0.200
3SUP	1.000	0.036	<0.001	0.014	<0.001	<0.001	0.009	<0.001	1.000	1.000
3INF	1.000	<0.001	0.012	<0.001	0.027	0.029	0.025	1.000	1.000	1.000
3NASAL	1.000	0.731	<0.001	<0.001	1.000	<0.001	0.089	0.017	1.000	0.007
3TEMP	1.000	<0.001	<0.001	<0.001	0.098	<0.001	0.054	<0.001	1.000	<0.001
6SUP	1.000	<0.001	<0.001	<0.001	<0.001	<0.001	<0.001	<0.001	1.000	0.009
6INF	1.000	<0.001	<0.001	0.001	<0.001	<0.001	0.002	<0.001	1.000	<0.001
6NASAL	0.832	<0.001	<0.001	<0.001	<0.001	<0.001	<0.001	<0.001	1.000	<0.001
6TEMP	0.634	0.006	<0.001	<0.001	<0.001	<0.001	<0.001	<0.001	1.000	<0.001
MV	0.232	<0.001	<0.001	<0.001	<0.001	<0.001	<0.001	<0.001	1.000	<0.001
AVERAGE	0.254	<0.001	<0.001	<0.001	<0.001	<0.001	<0.001	<0.001	1.000	<0.001

**Table 2:** *p*-values given by Wilcoxon test of comparison between operated and unoperated eyes. Differences with *p*-values < 0.05 are shown in shade.

Variable	Pre	7d	30d	90d	180d
CENTRAL	0.166	1.000	1.000	0.398	0.452
3SUP	1.000	1.000	0.328	0.029	0.035
3INF	1.000	1.000	1.000	0.053	0.049
3NASAL	0.235	1.000	1.000	0.032	0.013
3TEMP	1.000	1.000	1.000	0.107	0.273
6SUP	1.000	1.000	1.000	0.480	0.772
6INF	1.000	0.686	0.035	0.124	0.030
6NASAL	1.000	0.449	0.052	0.006	0.005
6TEMP	1.000	1.000	1.000	0.079	0.053
MV	1.000	1.000	0.487	0.007	0.007
AVERAGE	1.000	1.000	0.392	0.009	0.005

## Data Availability

All the data generated or analyzed during this study are included in this article. Further enquiries can be directed to the corresponding author.

## Supplementary Materials

The following supporting information can be downloaded at: https://www.mdpi.com/article/10.3390/diagnostics12123078/s1, Table S1: Preoperative and postoperative macular thicknesses and volumes (diabetic and non-diabetic patients) using Cirrus HD optical coherence tomography.
