# Supplementary material for: Evaluation of Macular Thickness Changes after Uncomplicated Phacoemulsification Surgery in Healthy Subjects and Diabetic Patients without Retinopathy by Spectral Domain OCT
Source: Diagnostics (Basel). 2022 Dec 7;12(12):3078. doi: 10.3390/diagnostics12123078 (PMC9776892; doi:10.3390/diagnostics12123078)
Supplement: Supplementary file 1 [file diagnostics-12-03078-s001.zip › diagnostics-2016639-supplementary.pdf]

**Table S1.** Preoperative and postoperative macular thicknesses and volumes (diabetic and non-diabetic patients) using Cirrus HD optical coherence tomography. Values are expressed as means, standard deviations (in parentheses) and 95% confidence intervals.

| | Preoperative | 1 week | 1 month | 3 months | 6 months |
|---|---|---|---|---|---|
| CENTRAL | 264.90 (22.64)<br>(259.25, 270.56) | 263,40 (21.44)<br>(258.04,268.75) | 268,80 (23.62)<br>(262.90, 274.70) | 273,95 (24.08)<br>(267.93, 279.96) | 269,76 (22.27)<br>(264.19, 275.32) |
| 3SUP | 320,00 (21.99)<br>(314.51, 325,50) | 322,18 (19.26)<br>(317.37, 327,00) | 325,59 (19.43)<br>(320.74, 330.45) | 329.93 (19.68)<br>(325.02, 334.85) | 328.09 (21.14)<br>(322.81, 333.38) |
| 3INF | 320.63 (16.84)<br>(316.42, 324.83) | 316.63 (24.32)<br>(310.56, 322.71) | 324.70 (16.75)<br>(320.52, 328.89) | 326.88 (18.81)<br>(322.18, 331.57) | 325.65 (16.62)<br>(321.50, 329.80) |
| 3NASAL | 325.78 (17.13)<br>(321.50, 330.06) | 325.09 (18.46)<br>(320.48, 329.71) | 328.73 (19.89)<br>(323.76, 330.70) | 334.03 (18.02)<br>(329.53, 338.53) | 330.81 (17.25)<br>(326.50, 335.12) |
| 3TEMP | 312.59 (16.70)<br>(308.42, 316.76) | 311.45 (17.97)<br>(306.97, 315.94) | 315.91 (16.65)<br>(311.75, 320.07) | 319.74 (17.05)<br>(315.48, 324.00) | 317.00 (16.70)<br>(312,83, 321,17) |
| 6SUP | 277.32 (14.62)<br>(273.67, 280.97) | 277.65 (14.94)<br>(273.92, 281.39) | 281.61 (15.35)<br>(277.78, 285.44) | 285.06 (14.75)<br>(281.38, 288.74) | 282.10 (15.52)<br>(278.22, 285.98) |
| 6INF | 267.39 (15.35)<br>(263.56, 271.23) | 268.06 (15.13)<br>(264.28, 271.83) | 271.18 (15.65)<br>(267.27, 275.09) | 274.15 (14.35)<br>(270.57, 277.74) | 270.77 (13.68)<br>(267.35, 274.18) |
| 6NASAL | 293.32 (15.15)<br>(289.54, 297.11) | 294.17 (16.02)<br>(290.17, 298.18) | 298.04 (15.97)<br>(294.05, 302.03) | 301.05 (15.62)<br>(297.15, 304.95) | 298.20 (15.43)<br>(294.35, 302.06) |
| 6TEMP | 263.27 (14.13)<br>(259.74, 266.80) | 262.35 (14.41)<br>(258.75, 265.95) | 265.59 (13.91)<br>(262.12, 269.07) | 269.72 (13.78)<br>(266.28, 273.16) | 266.36 (13.58)<br>(262.97, 269.76) |
| VOL | 10.06 (0.50)<br>(9.94, 10.19) | 10.10 (0.51)<br>(9.97, 10.23) | 10.22 (0.52)<br>(10.09, 10.35) | 10.34 (0.49)<br>(10.22, 10.46) | 10.23 (0.48)<br>(10.10, 10.35) |
| AVERAGE | 279.52 (13.67)<br>(276.11, 282.94) | 280.38 (14.07)<br>(276.86, 283.89) | 284.09 (14.36)<br>(280.50, 287.67) | 287.24 (13.69)<br>(283.82, 290.66) | 283.96 (13.40)<br>(280.62, 387.31) |

The thickness measurements are expressed in microns and volume in $mm^3$. Abbreviations: CENTRAL is the central 1mm ring; 3SUP, 3INF, 3NASAL, 3TEMP are, respectively, the superior, inferior, nasal and temporal quadrants of the 3 mm ring in the ETDRS grid; 6SUP, 6INF, 6NASAL, 6TEMP are, respectively, the superior, inferior, nasal and temporal quadrants of the 6 mm ring in the ETDRS grid; MV is the macular volume and AVERAGE is the average retinal thickness.
